# Supplementary material for: Molecular Detection and Phylogenetic Analyses of Babesia spp. and Theileria spp. in Livestock in Bangladesh
Source: Microorganisms. 2023 Jun 13;11(6):1563. doi: 10.3390/microorganisms11061563 (PMC10301362; doi:10.3390/microorganisms11061563)
Supplement: Supplementary file 1 [file microorganisms-11-01563-s001.zip › Table S1.pdf]

Table S1. prevalence of piroplasms according to the sex of the host animals

Pathogens	Animal species						Total
	Cattle		Goats		Gayals		
	Male (n=46)	Female (n=128)	Male (n=24)	Female (n=69)	Male (n=3)	Female (n=6)	
B. bigemina	30 (65.22%)	65 (50.78%)	6 (25.00%)	34 (49.28%)	n.d.	1 (16.67%)	136 (49.28%)
B. bovis	n.d.	1 (0.78%)	n.d.	1 (1.45%)	n.d.	n.d.	2 (0.72%)
B. naoakii	1 (2.17%)	n.d.	n.d.	2 (2.90%)	n.d.	n.d.	3 (1.09%)
*B. ovis**	n.s.	n.s.	8 (33.33%)	22 (31.88%)	n.s.	n.s.	30 (32.26%)
T. annulata	1 (2.17%)	8 (6.25%)	n.d.	2 (2.90%)	2 (66.66%)	5 (83.33%)	18 (6.52%)
T. orientalis	34 (73.91%)	83 (64.84%)	1 (4.17%)	6 (8.70)	1 (33.33%)	3 (50.00%)	128 (46.01%)

*Only screened for goats (n=93); n.d. not detected; n.s. not screened
