# Supplementary material for: Molecular Detection and Phylogenetic Analyses of Babesia spp. and Theileria spp. in Livestock in Bangladesh
Source: Microorganisms. 2023 Jun 13;11(6):1563. doi: 10.3390/microorganisms11061563 (PMC10301362; doi:10.3390/microorganisms11061563)
Supplement: Supplementary file 1 [file microorganisms-11-01563-s001.zip › Table S2.pdf]

Table S2. Co–infections with piroplasms in the blood samples of livestock in Bangladesh

Deleted: 1

Status of infection	Jhenaidah (n = 29)	Bogura (n = 14)	Sirajganj (n = 107)	Bandarban (n = 105)	Naikhonchari (n = 21)	Total (%) (n=276)
**Single infection**						
B. bigemina	3 (10.34%)	4 (28.57%)	29 (27.10%)	3 (2.86%)	n.d.	39 (14.13)
B. ovis	3 (10.34%)	n.d.	11 (10.28%)	n.d.		14 (5.07)
T. annulata	n.d.	n.d.	n.d.	1 (0.95%)	6 (28.57%)	7 (2.54)
T. orientalis	4 (13.79%)	3 (21.43%)	3 (2.80%)	23 (21.90)	1 (4.76%)	34 (12.32)
Sub total	10 (34.48%)	7 (50.00%)	43 (40.19%)	27 (25.71%)	7 (33.33%)	94 (34.04)
**Double infections**						
B. bigemina + *B. naoakii*	n.d.	n.d.	1 (0.93%)	n.d.	n.d.	1 (0.36)
B. bigemina + *B. ovis*	3 (10.34%)	n.d.	8 (7.48%)	1 (0.95%)	n.d.	12 (4.35)
B. naoakii + *B. ovis*	n.d.	n.d.	1 (0.93%)	n.d.	n.d.	1 (0.36)
B. bigemina + *T. annulata*	n.d.	n.d.	n.d.	1 (0.95%)	n.d.	1 (0.36)
B. bigemina + *T. orientalis*	3 (10.34%)	4 (28.57%)	26 (24.30%)	46 (43.81%)	n.d.	79 (28.62%)
B. bovis + *T. orientalis*	n.d.	n.d.	n.d.	1 (0.95%)	n.d.	1 (0.36)
B. ovis + *T. orientalis*	n.d.	n.d.	2 (1.87%)	n.d.	n.d.	2 (0.72)
T. annulata + *T. orientalis*	n.d.	n.d.	n.d.	1 (0.95%)	6 (28.57%)	7 (2.54)
Sub total	6 (20.68%)	4 (28.57%)	38 (35.51%)	50 (47.62%)	6 (28.57%)	104 (37.68)
**Triple infections**						
B. bigemina + *B. bovis* + *B. ovis*	n.d.	n.d.	1 (0.93%)	n.d.	n.d.	1 (0.36)
B. bigemina + *B. naoakii* + *T. orientalis*	n.d.	n.d.	1 (0.93%)	n.d.	n.d.	1 (0.36)
B. bigemina + *T. annulata* + *T. orientalis*	n.d.	n.d.	n.d.	1 (0.95%)	2 (9.52%)	3 (1.09)
Sub total	n.d.	n.d.	2 (1.87%)	1 (0.95%)	2 (9.52%)	5 (1.81)
Grand total	16 (55.17%)	11 (78.57%)	83 (77.57%)	78 (74.29%)	15 (71.43%)	203 (73.55)

n.d.: not detected
