# Supplementary figures and images for: Molecular Detection and Phylogenetic Analyses of Babesia spp. and Theileria spp. in Livestock in Bangladesh
Source: Microorganisms. 2023 Jun 13;11(6):1563. doi: 10.3390/microorganisms11061563 (PMC10301362; doi:10.3390/microorganisms11061563)

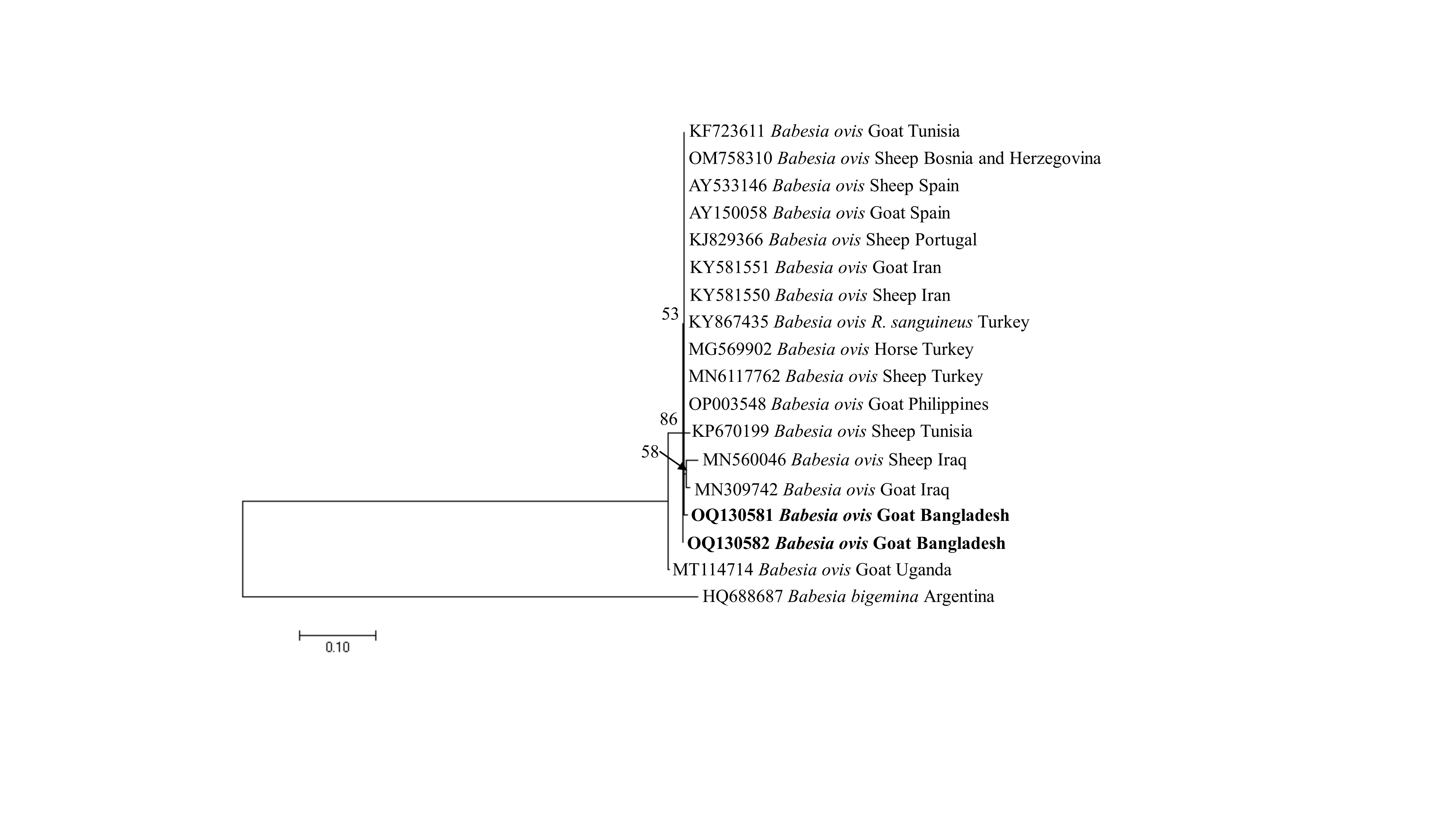

Supplement: Supplementary file 1 [file microorganisms-11-01563-s001.zip › Figure S1.tif]

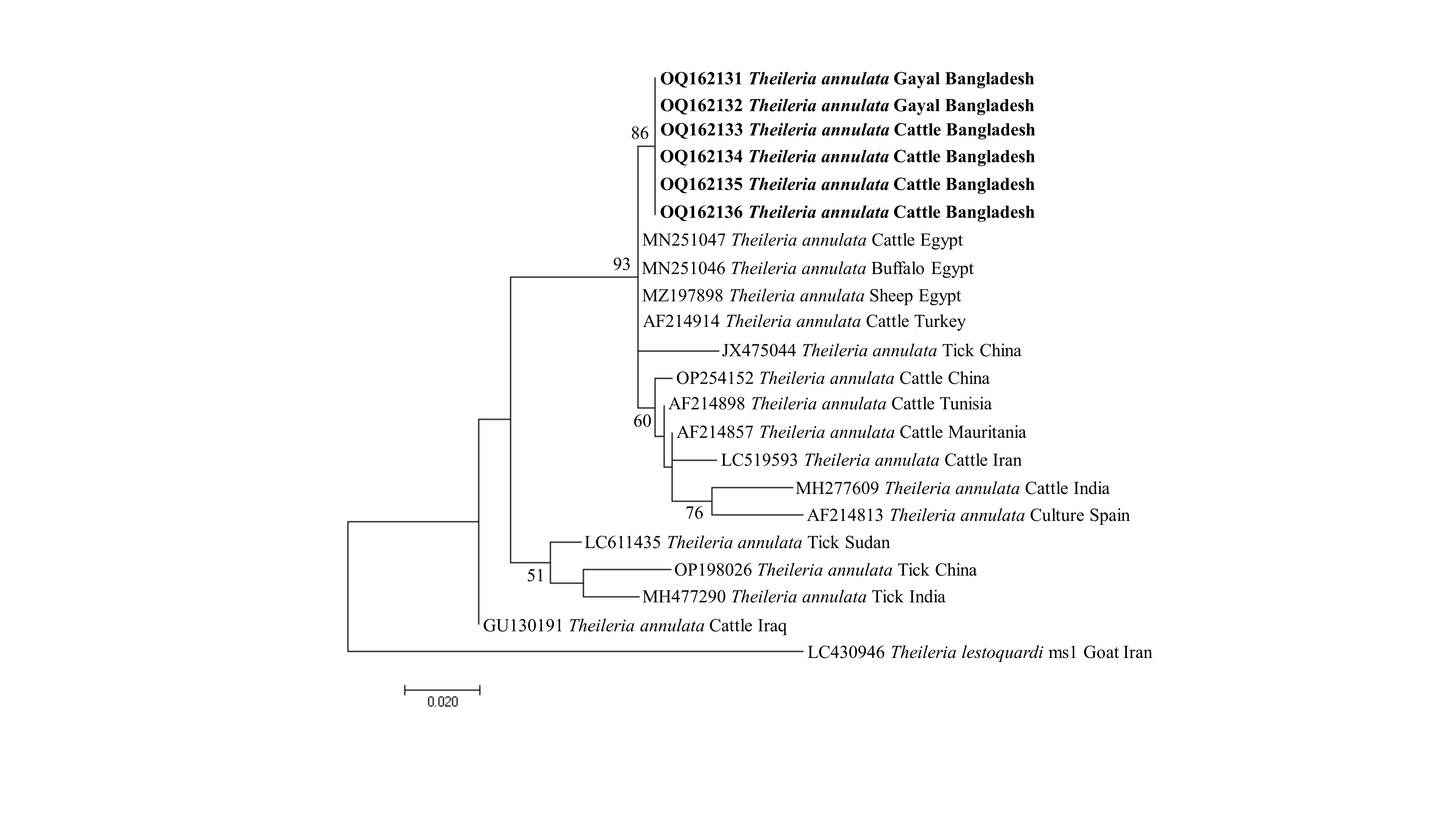

Supplement: Supplementary file 1 [file microorganisms-11-01563-s001.zip › Figure S2.tif]
